# Supplementary figures and images for: Investigation of the validity of two Bayesian ancestral state reconstruction models for estimating Salmonella transmission during outbreaks
Source: PLoS One. 2019 Jul 22;14(7):e0214169. doi: 10.1371/journal.pone.0214169 (PMC6645465; doi:10.1371/journal.pone.0214169)

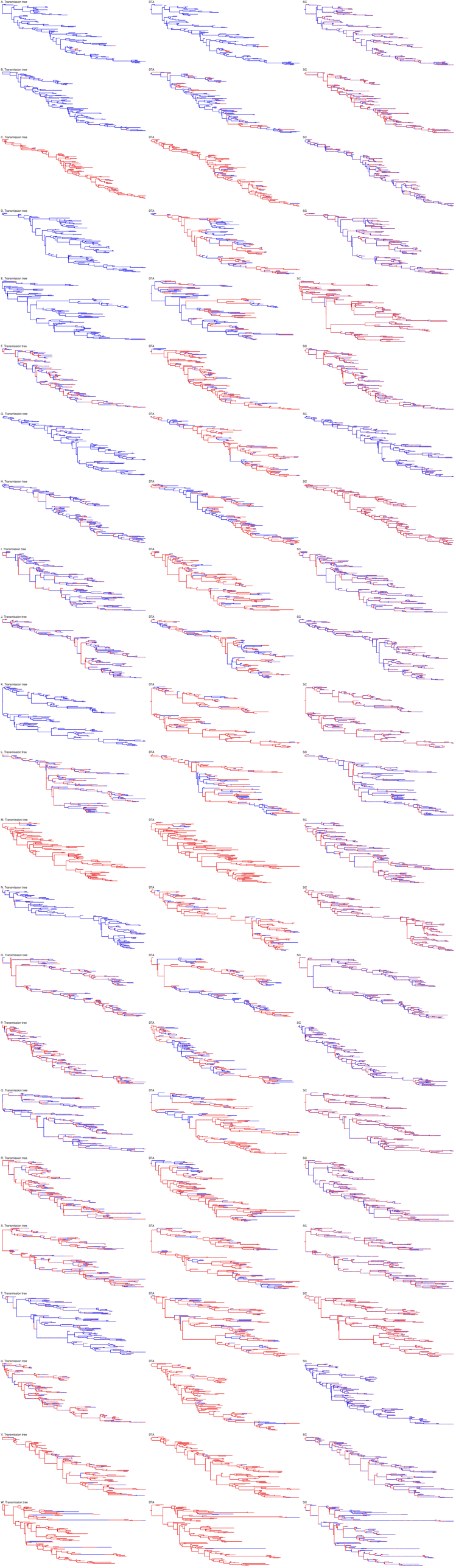

Supplement: S1 Fig — Sampled transmission trees, maximum clade credibility trees produced by the DTA model and maximum a posteriori trees produced by the SC model, for simulated outbreaks 1–23 (A-W, respectively) that 100 isolates were randomly sampled from. The blue areas represent time spent in the human population and the red areas represent time spent in the animal population. (PNG) [file pone.0214169.s002.png]

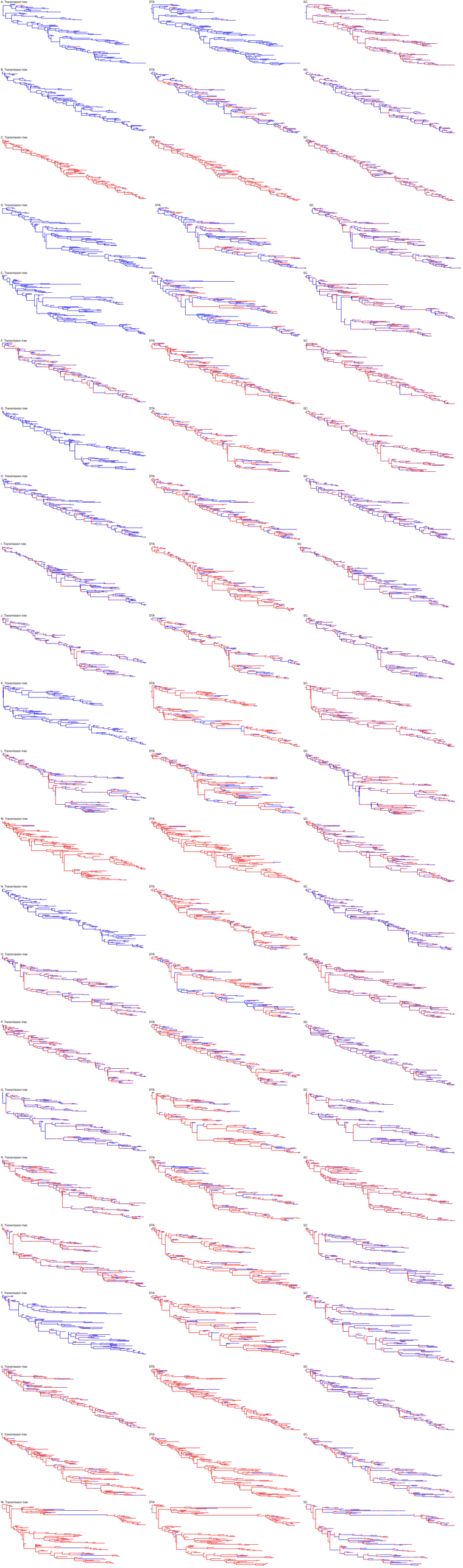

Supplement: S2 Fig — Sampled transmission trees, maximum clade credibility trees produced by the DTA model and maximum a posteriori trees produced by the SC model, for simulated outbreaks 1–23 (A-W, respectively) that 100 isolates were sampled equally over time. The blue areas represent time spent in the human population and the red areas represent time spent in the animal population. (PNG) [file pone.0214169.s003.png]

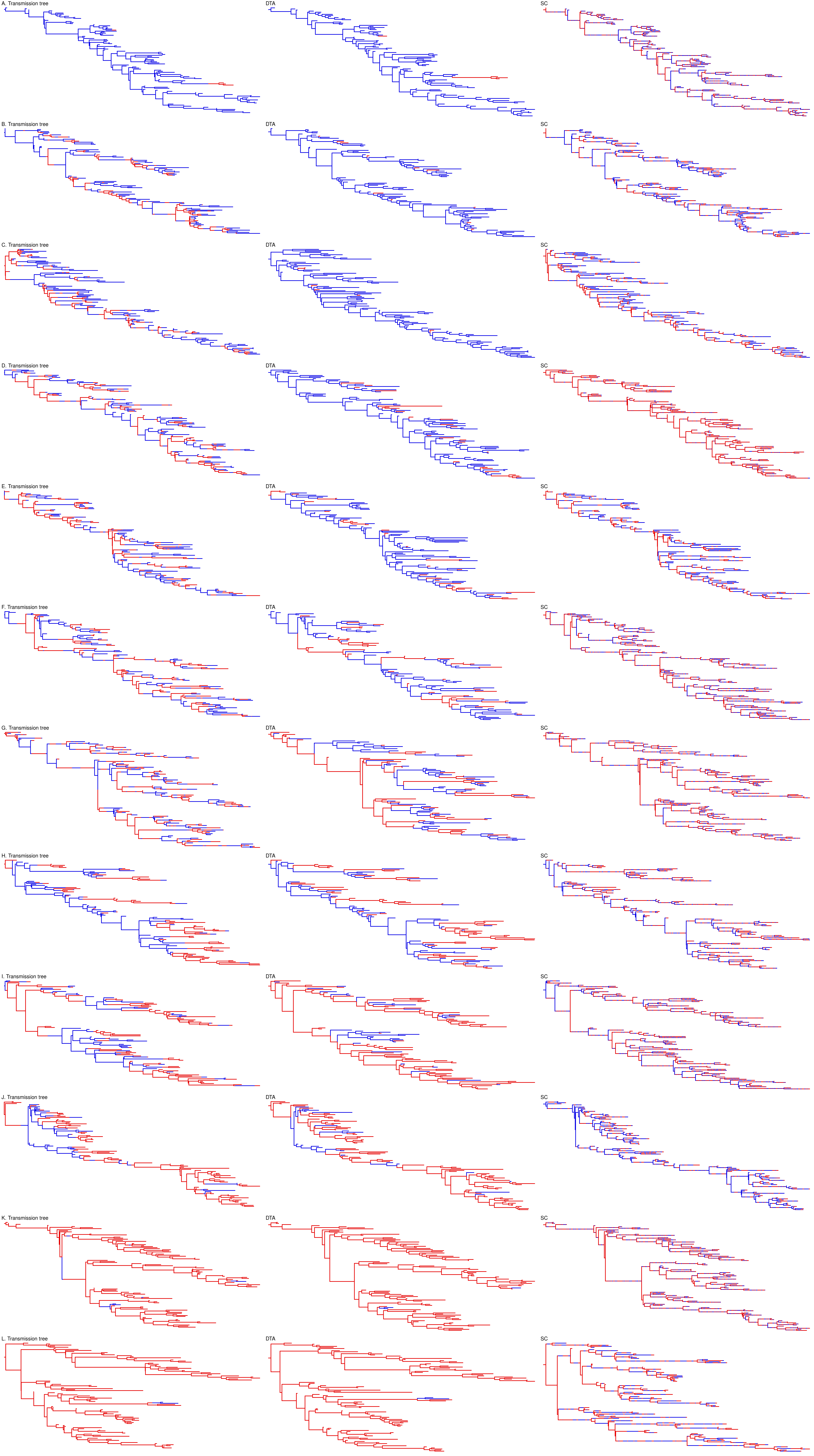

Supplement: S3 Fig — Sampled transmission trees, maximum clade credibility trees produced by the DTA model and maximum a posteriori trees produced by the SC model, for EPTI simulated outbreaks 24–35 (A-L, respectively) that 100 isolates were randomly sampled from. The blue areas represent time spent in the human population and the red areas represent time spent in the animal population. (PNG) [file pone.0214169.s004.png]

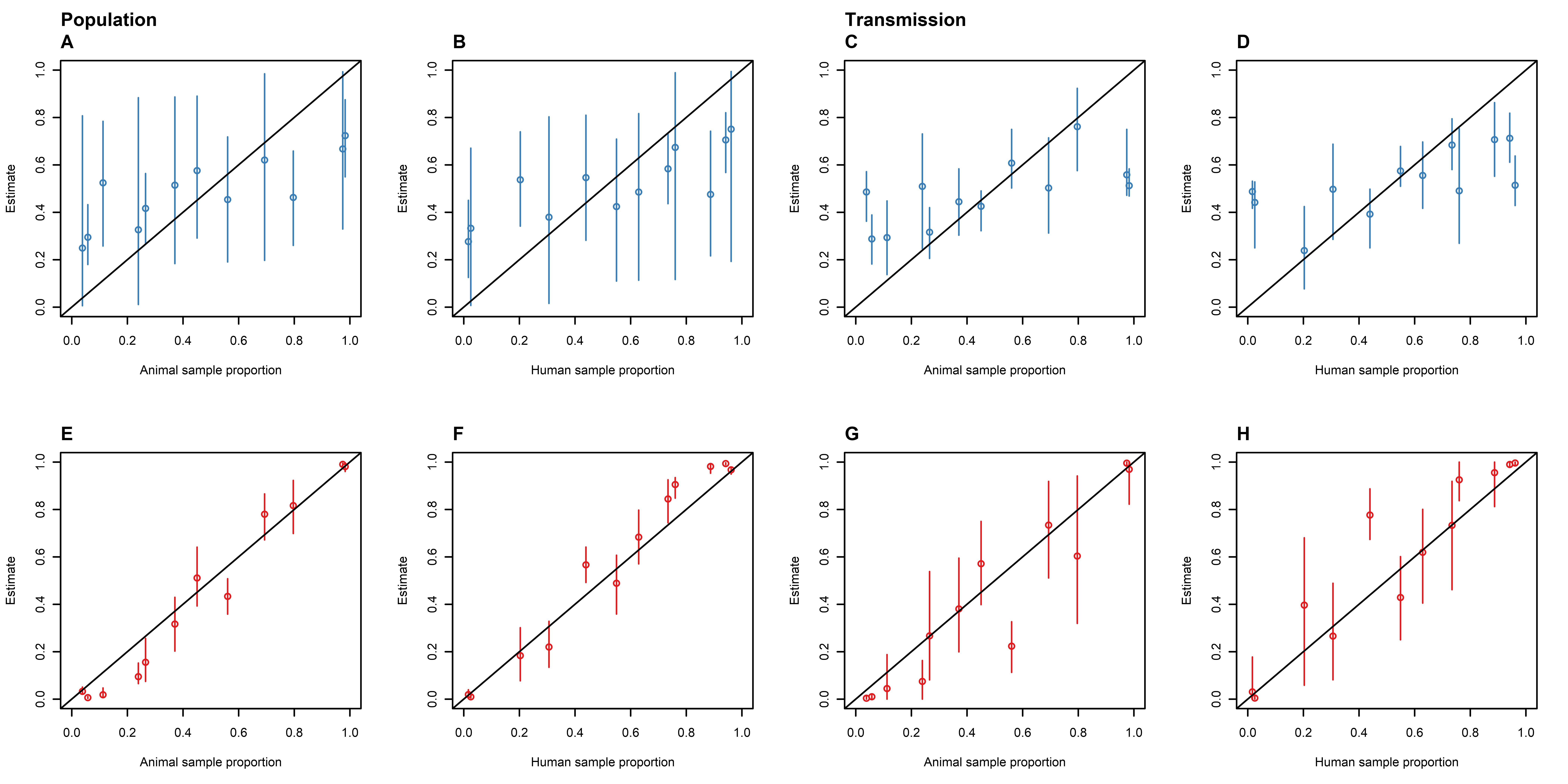

Supplement: S4 Fig — The proportion of time spent in the animal (A and E) and human (B and F) host populations, and the proportion of inter-population transmissions made up of animal-to-human (C and G) and human-to-animal (D and H) transmissions as estimated by the SC (blue: A-D) and DTA (red: E-F) models versus the proportion of samples made up of animal (A, C, E and G) and human (B, D, F and H) host populations for 12 EPTI simulated outbreaks that 100 isolates were randomly sampled from. The dots represent the mean, and the error bars represent the 95% HPD interval. (PNG) [file pone.0214169.s005.png]

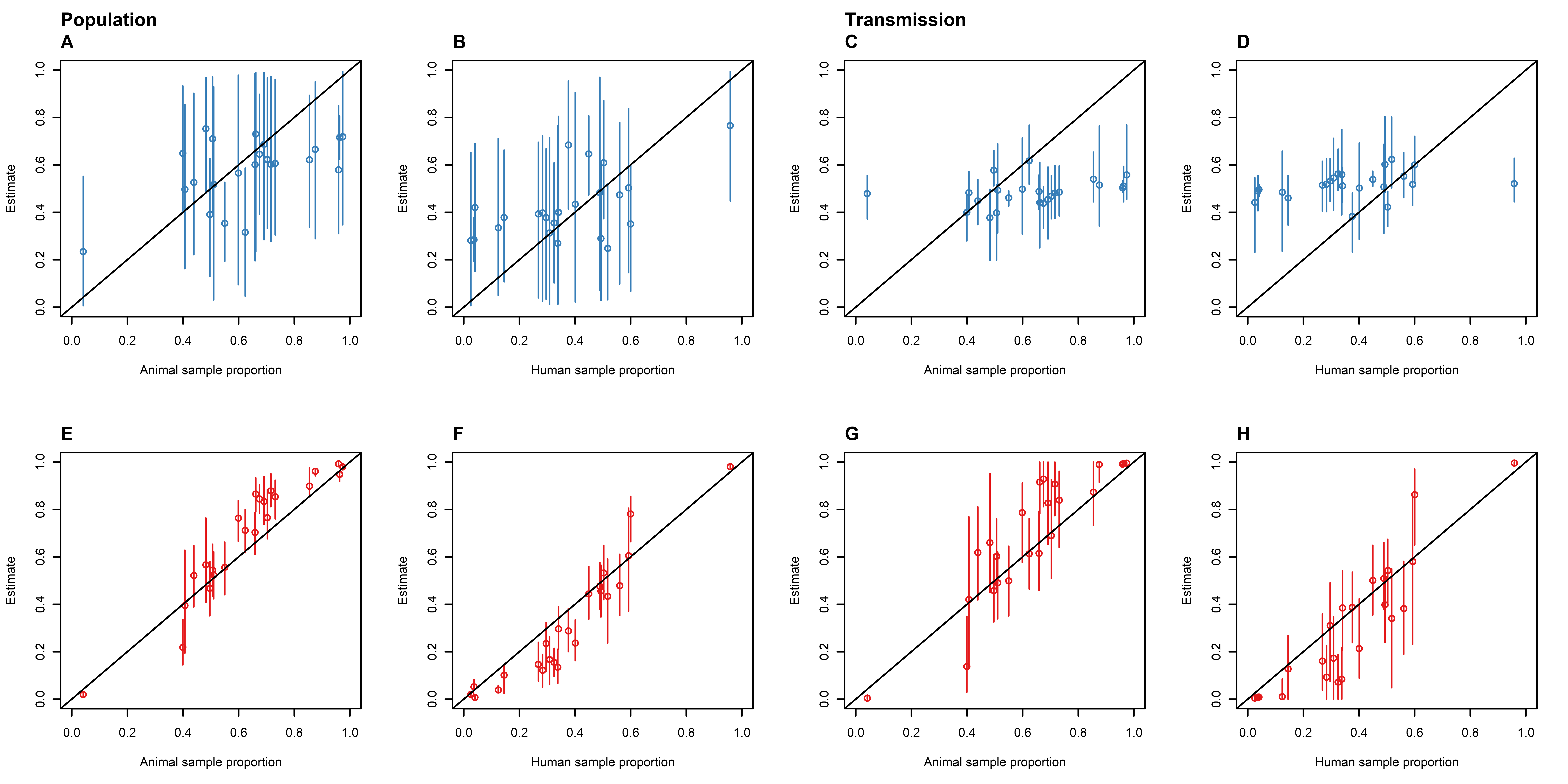

Supplement: S5 Fig — The proportion of time spent in the animal (A and E) and human (B and F) host populations, and the proportion of inter-population transmissions made up of animal-to-human (C and G) and human-to-animal (D and H) transmissions as estimated by the SC (blue: A-D) and DTA (red: E-F) models versus the proportion of samples made up of animal (A, C, E and G) and human (B, D, F and H) host populations for 23 simulated outbreaks that 100 isolates were randomly sampled from. The dots represent the mean, and the error bars represent the 95% HPD interval. (PNG) [file pone.0214169.s006.png]

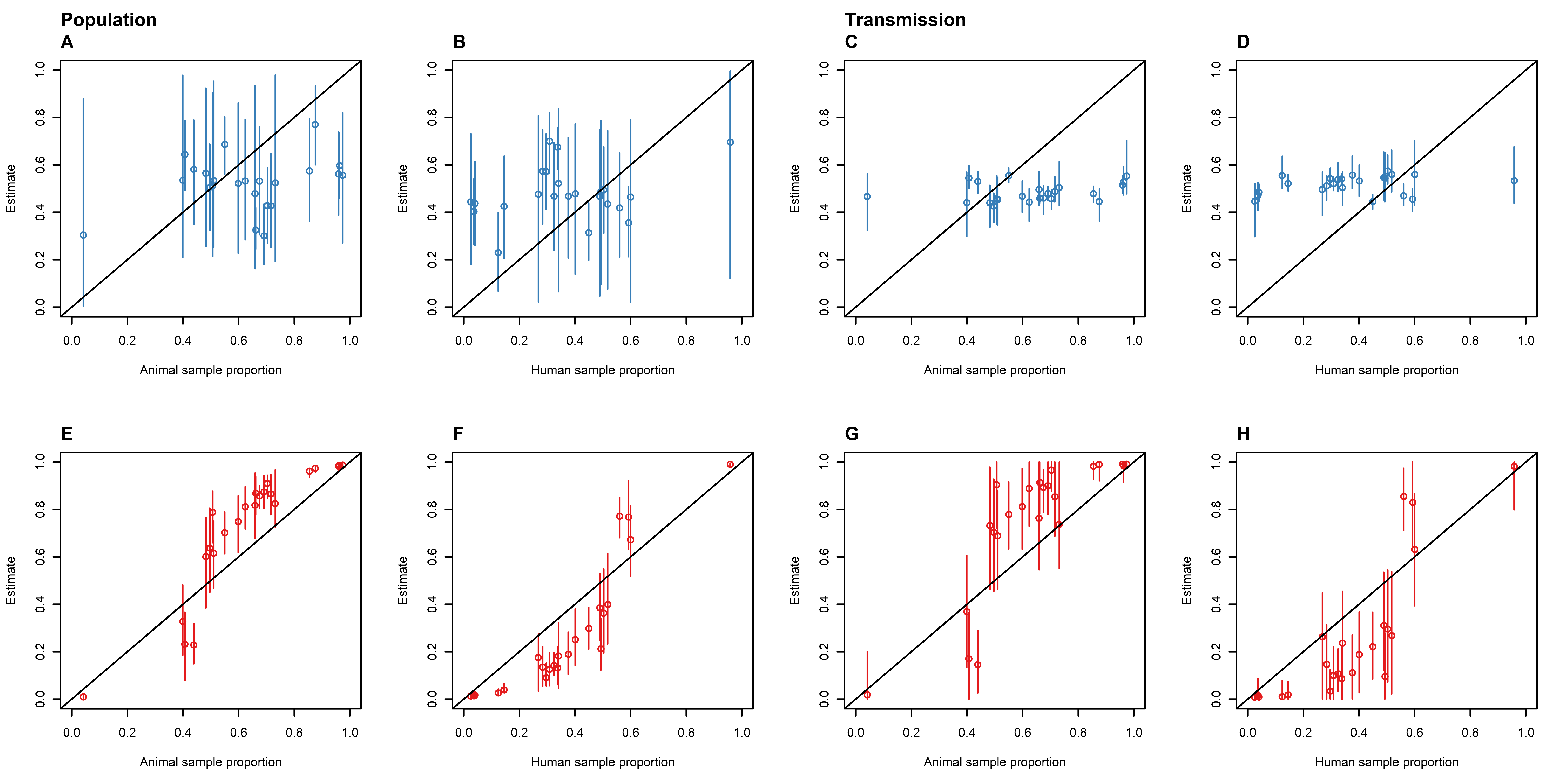

Supplement: S6 Fig — Scatterplots of the proportion of time spent in the animal (A and E) and human (B and F) host populations, and the proportion of inter-population transmissions made up of animal-to-human (C and G) and human-to-animal (D and H) transmissions as estimated by the SC (blue: A-D) and DTA (red: E-F) models versus the proportion of samples made up of animal (A, C, E and G) and human (B, D, F and H) host populations for 23 simulated outbreaks that 100 isolates were sampled equally over time from. The dots represent the mean, and the error bars represent the 95% HPD interval. (PNG) [file pone.0214169.s007.png]

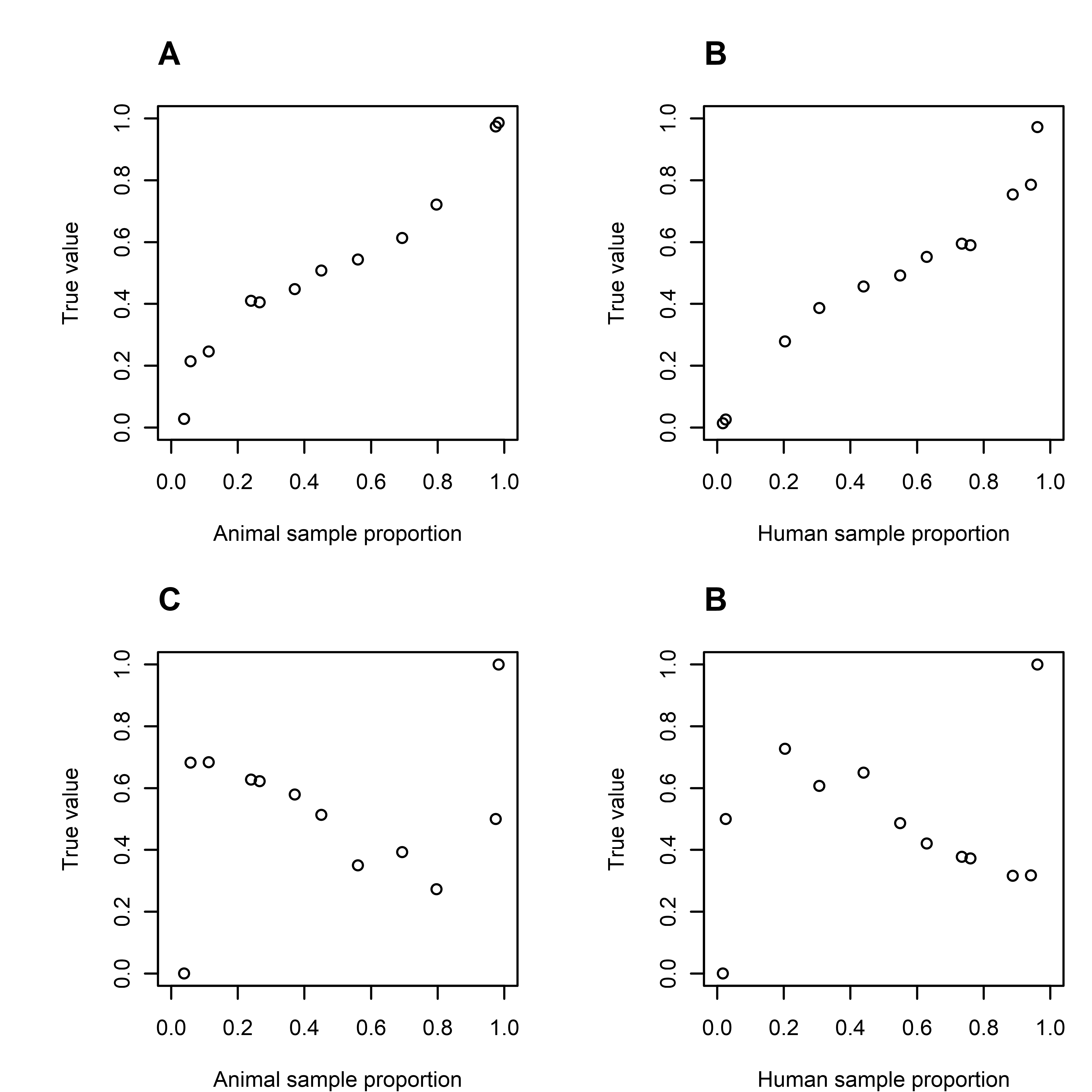

Supplement: S7 Fig — The proportion of samples made up of animal (A and C) and human (B and D) host populations, versus the known population (A and B) and transmission (C and D) parameters for 12 EPTI simulated outbreaks that 100 isolates were randomly sampled from. (PNG) [file pone.0214169.s008.png]

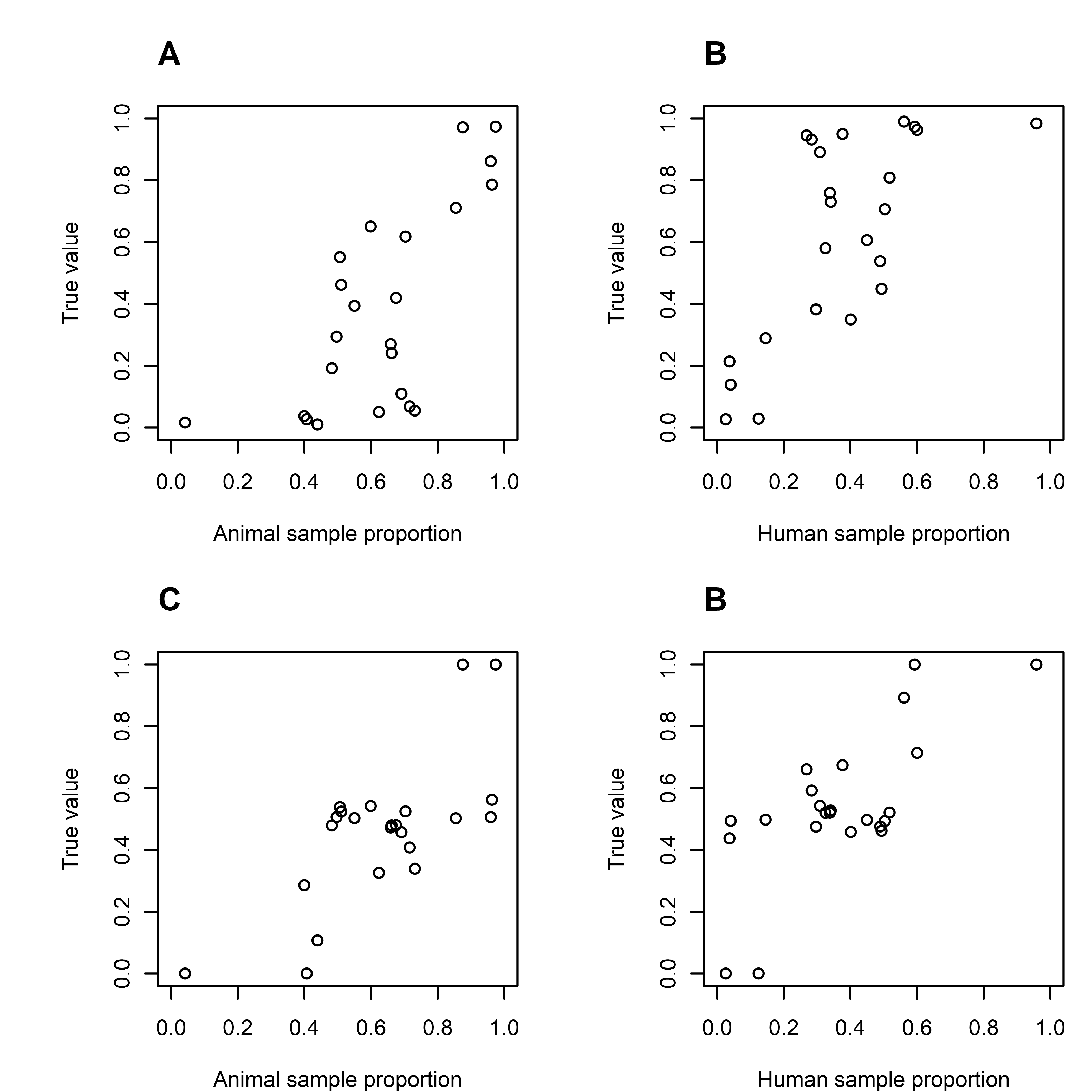

Supplement: S8 Fig — The proportion of samples made up of animal (A and C) and human (B and D) host populations, versus the known population (A and B) and transmission (C and D) parameters for 23 simulated outbreaks that 100 isolates were randomly sampled from. (PNG) [file pone.0214169.s009.png]

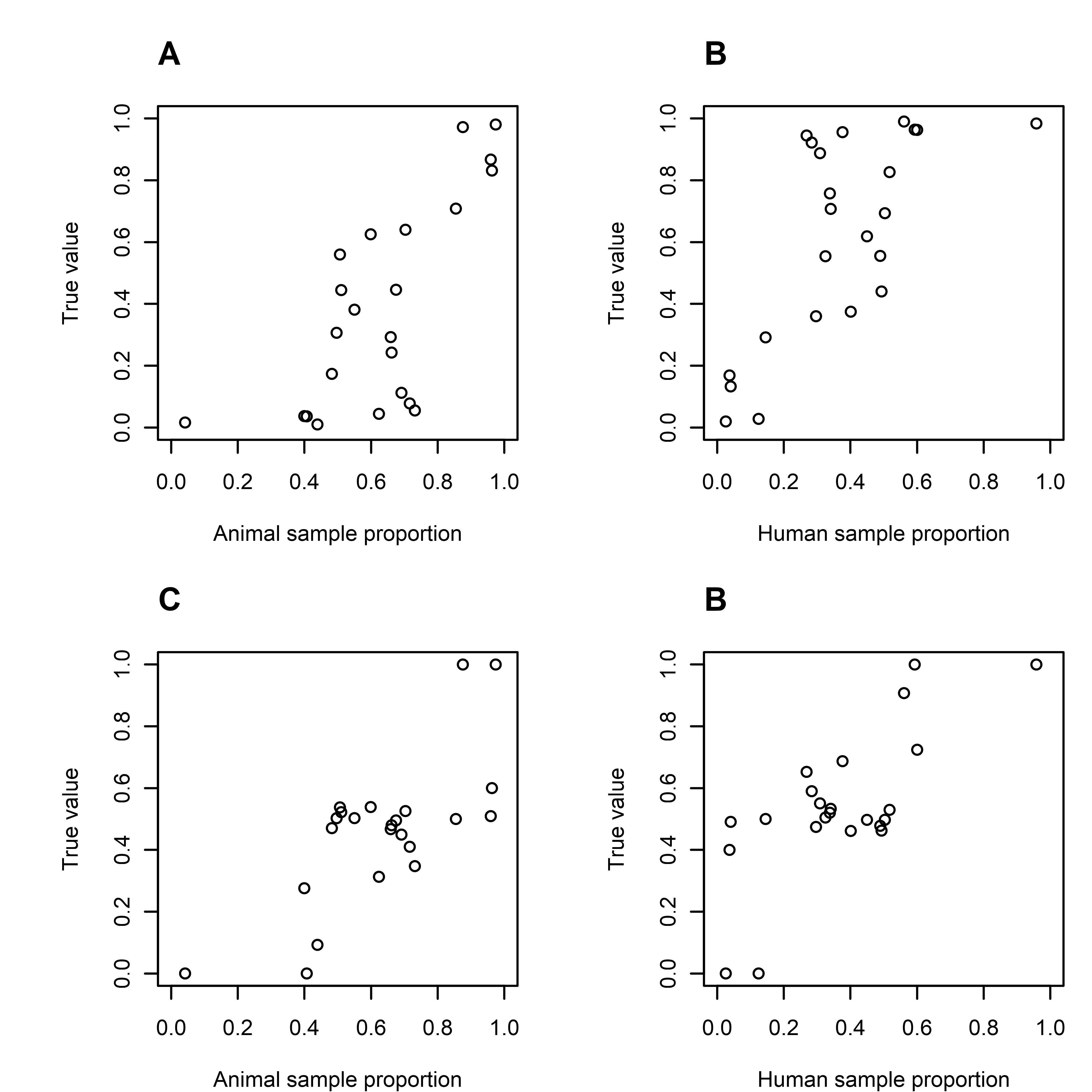

Supplement: S9 Fig — The proportion of samples made up of animal (A and C) and human (B and D) host populations, versus the known population (A and B) and transmission (C and D) parameters for 23 simulated outbreaks that 100 isolates were sampled equally over time from. (PNG) [file pone.0214169.s010.png]
